# Estimating the mutational fitness effects distribution during early HIV infection

**DOI:** 10.1093/ve/vey029

**Published:** 2018-10-04

**Authors:** Eva Bons, Frederic Bertels, Roland R Regoes

**Affiliations:** 1 Department of Environmental Systems Science, Institute of Integrative Biology, ETH Zurich, Universitätstrasse 16, Zurich, Switzerland; 2 Department for Evolutionary Theory, Max Planck Institute for Evolutionary Biology, August-Thienemann-Str. 2, Plön, Germany

**Keywords:** HIV, fitness effects distribution, primary infection, computational model, ABC-SMC

## Abstract

The evolution of HIV during acute infection is often considered a neutral process. Recent analysis of sequencing data from this stage of infection, however, showed high levels of shared mutations between independent viral populations. This suggests that selection might play a role in the early stages of HIV infection. We adapted an existing model for random evolution during acute HIV-infection to include selection. Simulations of this model were used to fit a global mutational fitness effects distribution to previously published sequencing data of the *env* gene of individuals with acute HIV infection. Measures of sharing between viral populations were used as summary statistics to compare the data to the simulations. We confirm that evolution during acute infection is significantly different from neutral. The distribution of mutational fitness effects is best fit by a distribution with a low, but significant fraction of beneficial mutations and a high fraction of deleterious mutations. While most mutations are neutral or deleterious in this model, about 5% of mutations are beneficial. These beneficial mutations will, on average, result in a small but significant increase in fitness. When assuming no epistasis, this indicates that, at the moment of transmission, HIV is near, but not on the fitness peak for early infection.

## 1. Introduction

Evolution is driven by new mutations that cause a change in fitness of the organism. If a new mutation increases the reproductional success, or the fitness, then this mutation is likely to be selected for and eventually fix in a population. However, most mutations are not beneficial to the organism. Instead they are neutral—having no effect on fitness, or deleterious, reducing the amount of offspring compared with the ancestor. The effects a mutation can have on fitness lie on a continuum from completely lethal to beneficial, including viable but deleterious effects and neutral effects. The mutational fitness effects distribution (MFED, reviewed by Eyre-Walker and Keightley (2007)) captures how these effects are distributed for a certain organism in a certain environment.

The MFED has been inferred for several viruses using site-directed mutagenesis studies (Sanjuán 2010) and deep sequencing of cultured virus (Acevedo, Brodsky, and Andino 2014). It has a similar shape across different virus species, with a sizable fraction of mutations (20%–40%) being lethal and the rest forming a single peak at or close to zero. In some, but not all of these MFEDs, a small fraction (<10%) of beneficial mutations is observed. Knowing the MFED of an organism in a certain environment can help us understand and predict the evolutionary dynamics of this organism in this environment. But the concept of the MFED has its limitations, in that it assumes global fitness effects of mutations. Because fitness depends on the environment, and environments can change over time, the insights into the MFED from one environment to another have to be transferred with caution.

Early HIV infection is characterized by a rapid expansion of the virus population. Transmission is a bottleneck, as only one to five viruses are responsible for the establishment of an infection (Keele et al. 2008). After a few weeks, virus levels can reach up to 10^6^ virus particles per ml plasma (Fiebig et al. 2003). This expansion is accompanied by rapid sequence diversification due to the high mutation rate of HIV. This rate is estimated to be between 1.1×10^–5^ and 5.8×10^–3^ mutations per base per replication, depending on the method and source material of the estimation (Mansky and Temin 1995; Huang and Wooley 2005; Dapp, Heineman, and Mansky 2013; Cuevas et al. 2015). The very high mutation rate estimated by Cuevas et al. is likely due to the activity of APOBEC3G, a human protein that causes G-to-A hypermutation in certain sequence motives. In most cases where APOBEC proteins are active, they introduce many mutations in a single sequence, often leading to the introduction of a stop-codon which leads to non-viable virus. HIV is protected from APOBEC hypermutation by its vif-protein, so that in some cases, APOBEC only introduces a few mutations resulting in viable virus with more G-to-A mutations in the specific APOBEC-sequence context than expected. The exact activity of APOBEC-proteins depends on their expression level in the infected cell (Huang and Wooley 2005), and the activity of the viral protein vif (Simon et al. 2005), causing great variation in the probability of observing APOBEC-mediated mutations across HIV-infected individuals.

During these early stages of infection, HIV evolution is often considered a neutral process due to the rapid expansion of the virus population and absence of immune response, which is the main evolutionary pressure on HIV during infection. Keele et al. (2008) and Lee et al. (2009) showed that sequence patterns in the *env* gene of eighty-two individuals show typical signs of neutral evolution during acute infection, such as a star-like phylogeny. While the mutational patterns appear neutral on an individual level, signs of selection become clear when looking at the viral populations in several individuals at once. Wood et al. (2009) and Bertels, Metzner, and Regoes (2018) studied the same dataset and found several convergent mutations; mutations that appear in several viral populations independently. These mutations are likely positively selected, indicating evolution during early HIV infection is not neutral.

By estimating the MFED for HIV during early infection, evolutionary pressures during these early stages of infection can be better understood. There have been several attempts at estimating fitness effects of mutations for HIV, but they either only consider the amino-acid level (Ferguson et al. 2013; Haddox, Dingens, and Bloom 2016) or analyze a subset of mutations only, such as deleterious mutations (Zanini et al. 2017) or resistance mutations (Martinez-Picado and Martínez 2008). Because the immune response exerts temporally varying, potential strong selection pressure on viral populations (Fernandez et al. 2005; Asquith et al. 2006; Mandl et al. 2007; Regoes et al. 2007; Regoes, Yates, and Antia 2007; Ganusov et al. 2011; Bar et al. 2012; Kessinger, Perelson, and Neher 2013), it is important to focus on the very early phases of infection before adaptive immune responses are mounted.

In this paper, we use these patterns of convergent evolution found by Wood et al. (2009) and Bertels, Metzner, and Regoes (2018) to estimate the a global MFED of HIV during early infection. For this, we developed a simulation model of viral dynamics and sequence evolution, which allows viral strains to differ in their fitness. This simulation model was fitted to patterns of sharing in *env* sequences collected by Keele et al. (2008) and Li et al. (2010).

## 2. Results

### 2.1 Simulation of the molecular evolution of HIV during early infection

To investigate the impact of viral fitness differences on HIV sequence evolution in infected individuals, we developed a simulation model of sequence evolution in early HIV infection.

The model is based on the Monte Carlo simulations of the synchronous infection model for HIV sequence evolution presented in Lee et al. (2009). In contrast to Lee et al., however, we relax the assumption that mutations are neutral. Instead, we assign a fitness advantage or disadvantage to each mutation that occurs according to a mutational effects distribution. We parameterized this distribution fairly flexibly, such that it describes a fraction of beneficial, detrimental and completely lethal effects, and can also be collapsed, for appropriate parameters, to a fully neutral model (as in Lee et al.).

A simulation starts with a randomly generated sequence with a relative fitness of one. In every generation, all sequences in the population generate *N_s_* new offspring. Point mutations can occur in these offspring sequences, which can alter the fitness of the sequence. *N_s_* is a random number drawn from a Poisson distribution with mean $R_{0}\cdot f$. In this formula, *R*_0_ denotes the absolute fitness of the virus, which we set to 6 in accordance with Lee et al. (2009), and *f* is the relative fitness compared with the ancestor sequence.

**Figure 1.**
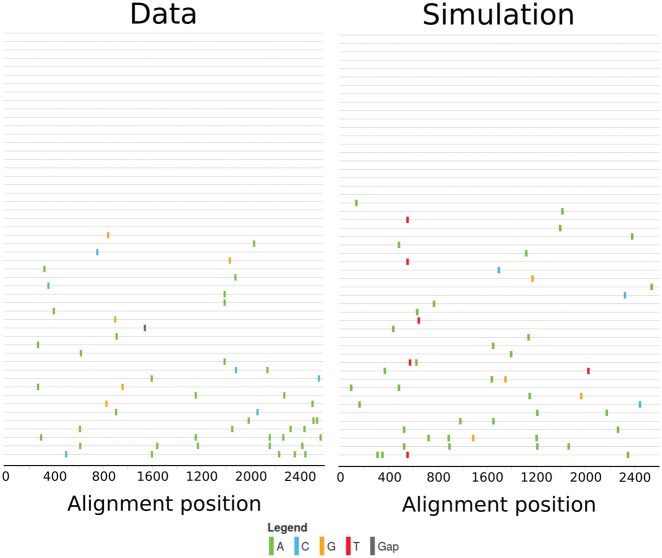
Visualization of sequence samples using highlighter (hiv.lanl.gov) of the sequence sample from subject 1,018 (left) and the output of a simulation matching this data (right). Both sequence samples consist of fifty-one sequences. In the empirical sample, there are fifty mutations, of which fourty-four occur only in a single viral population. Twenty-four sequences are unmutated. In the simulated sample, there are fourty-nine mutations, of which fourty-four occur only in a single viral population and twenty sequences are unmutated.

Our goal with these simulations is to generate datasets of the same structure as the dataset by Keele et al. and Li et al. by matching the number of generations in our simulation to the time since infection (TSI) of the infected individual in the empirical datasets. All known estimates for the TSI, however, are based on a neutral model of evolution, which can lead to a bias. To avoid this potential bias in the TSI, simulations were run for as long as necessary to match the number of unique mutations in a sample of the same size as available in the data set. The resulting sequence sample (see Fig. 1) has similar mutational characteristics as the sequence sample available for each subject, without requiring to specify for how many generations the simulation should be run.

### 2.2 Estimating the shape of the mutational effects distribution

In order to calculate fitness of a mutated sequence, every possible mutation is assigned a fitness effect according to the MFED. Each of these effects is assumed to apply universally in all hosts, there are no host-specific effects. Total fitness of a sequence is then the product of the fitness effects of all mutations in the sequence.

**Figure 2.**
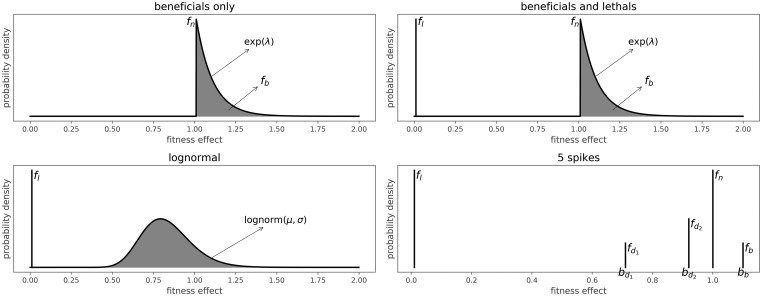
Overview of four of the six different models for the MFED. The beneficials only model has two free parameters (*fb*; *λ*), the beneficials and lethals model has three free parameters (*fl*; *fb*; *λ*), the log-normal model has three free parameters (*fl*; *μ*; *σ*). The 5 spikes model has seven free parameters (*fl*; *fd*1; *bd*1; *fd*2; *bd*2; *fb*; *bb*).

The effects in the MFED range from zero to infinity, with a fitness effect of one indicating a neutral mutation (see Fig. 2). Deleterious mutations have a fitness effect smaller than one, with an effect of zero indicating a lethal mutation. Sequences carrying a lethal mutation differ from sequences carrying a non-lethal deleterious mutation since they will never produce any offspring, while sequences carrying mutations with a very small, but not 0, fitness effect can still produce offspring, albeit with a very low probability. A very beneficial mutation might also compensate for such a deleterious mutation, while this is impossible in the case of lethal mutations.

The fitness effects distribution will affect the number of shared mutations across viral populations. While the probability of a mutation occurring does not change, the probability of a mutation being maintained in the population and later sampled is affected by the fitness effect of the mutation. Once a beneficial mutation occurs, the sequence carrying this mutation will create more offspring than unmutated sequences, and will be overrepresented in the viral population after a few generations. This increases the chance of observing the mutation in two or more samples.

Lethal or deleterious mutations will cause the sequence carrying the mutation to have fewer offspring and are therefore unlikely to be observed. Since these mutations are so unlikely to be observed, the sites can be considered immutable, which results in an effective shortening of the genome available for mutation. This might indirectly increase the chance of sharing mutations by increasing the chance that other, less detrimental, mutations are observed.

We defined six different models for the mutational effects distribution (see Fig. 2), as well as a neutral model where all fitness effects are one.

The first two models describe simplified distributions with a restricted effects range. The ‘beneficials only’ model consists only of neutral and beneficial fitness effects, and is defined by a fraction of beneficial mutational effects (*f_b_*), which are exponentially distributed with mean *λ*. The rest of the mutational effects are neutral ($f_{n}=1-f_{b}$). The second model, ‘lethals only’, consists of a fraction of lethal (*f_l_*, fitness effect of 0) and neutral effects ($f_{n}=1-f_{l}$). The third model is a combination of these, with a fraction of lethal mutations, a fraction of exponentially distributed beneficial mutations and the rest of the mutations is neutral. The last three models are more complex and try to capture a wider spectrum of conceivable fitness effects in order to replicate the observed distributions in other organisms mentioned in the Section 1. The log-normal model consists of log-normally distributed fitness effects, with parameters *μ* and *σ*, in the entire range from zero to infinity, plus a certain amount of fitness effects that are exactly 0, the fraction lethals (*f_l_*). A variation of this model is the ‘log-normal truncated’ model, which is the same as the log-normal model, only there are no beneficial mutations. Instead, all mutations that would have been beneficial in the log-normal model are now neutral. The last model, ‘5 spikes’, allows for exactly five different values for the fitness effects. Fitness effects can be zero (lethal), one (neutral), two values between 0 and 1 ($b_{d_{1}}$ and $b_{d_{2}}$, deleterious) or a value larger than 1 (*b_b_*, beneficial). The relative fraction of mutations with each effect are represented as *f_l_*, $f_{d_{1}},\;f_{d_{2}}$, *f_n_* and *f_b_*. This allows for an approximation of multi-modal models without more complex model definitions. The neutral model, where all mutations have a fitness effect of one is recovered by setting all parameters to zero in any of the models.

We use approximate Bayesian computation using sequential Monte Carlo (ABC-SMC) to do simultaneous model selection and parameter estimation. The SMC procedure starts with a set of parameters for all models, with equal amounts of parameter sets for each model. For each parameter set, a simulation is performed and those simulations whose distance to the data is below a threshold are accepted. In each subsequent iteration, new parameter sets are sampled from the set of accepted parameters in the previous iterations and the threshold for acceptance is lowered. This results in simultaneous parameter estimation and model selection, since a good model will have more parameter sets accepted, where bad models will have fewer accepted parameter sets. If no parameter sets are accepted for a certain model, this model has ‘died out’ and is considered a very unlikely model candidate.

This procedure requires summary statistics to calculate the distance between simulations and the data. The summary statistics used here (see Section 4 for a full overview) are based on measures of sharing, such as the distribution of the average degree of sharing of the mutations in a viral population and the number of populations the mutations appear in, and population-specific statistics such as inter-sequence hamming distance and the number of unmutated sequences in the sample were used.

### 2.3 The best estimate for the MFED

The model selection results in two equally likely models: the ‘lethals and beneficials’ model and the log-normal model. A summary of the parameter estimation and characteristics of the MFED with best-fit parameters for these models can be found in Table 1. Both models have a relative probability (‘support’) of approximately 0.4. The ‘beneficials only’ and ‘5 spikes’ model have a much lower support, both are <0.1. The neutral model, ‘lethals only’ model and the truncated log-normal model died out before the 4th iteration of the ABC-SMC.

**Table 1.** Fitted parameters with 95% HPD and moments of the MFED for the top two models.

	Lethals and beneficials	Log-normal
Parameters	*f_l_* = 0.182 (0.019; 0.552)	*f_l_* = 0.045 (0.003; 0.422)
	*f_b_* = 0.053 (0.008; 0.228)	*μ* = –0.248 (–0.284;–0.088)
	*β* = 0.202 (0.051; 0.732)	*σ* = 0.149 (0.104; 0.288)
	G →A = 10.451 (2.361; 69.169)	G →A = 10.621 (1.886; 74.797)
Support	0.439	0.409
Mean effect	0.828	0.753
Fraction beneficial	0.052	0.045
Beneficial effect	1.204	1.065
Beneficial gain	0.063	0.048
Fraction neutral	0.766	0
Fraction deleterious	0	0.909
Deleterious effect	0	0.776
Fraction lethal	0.182	0.046
Deleterious loss	0.182	0.250

For the top two models, enough simulations were accepted to use for parameter estimation. All parameters form a single peak (see Supplementary Fig. S3) in their posterior density distribution. Many parameters have a relatively large 95% highest probability density (HPD) interval, although all parameters are significantly different from the neutral value. The MFEDs corresponding to the best-fit models are very similar. Both distributions have a mean fitness effect of approximately 0.8. The log-normal model has slightly fewer beneficials (4.5% vs 5.2% in the ‘lethals and beneficials’ model) that also have a slightly lower effect. It is harder to compare the deleterious and lethal mutations between the two models, since the ‘lethals and beneficials’ model does not include deleterious mutations, while in the log-normal model, 90% of mutations are deleterious, albeit with a low effect. The deleterious loss (the product of the fraction of deleterious/lethal mutations and their loss of fitness, which equals 1 minus the fitness effect) is the easiest way to compare them. Although the ‘lethals and beneficials’ model has more lethal mutations, the large amount of deleterious mutations leads to an overall higher deleterious loss in the log-normal model.

**Figure 3.**
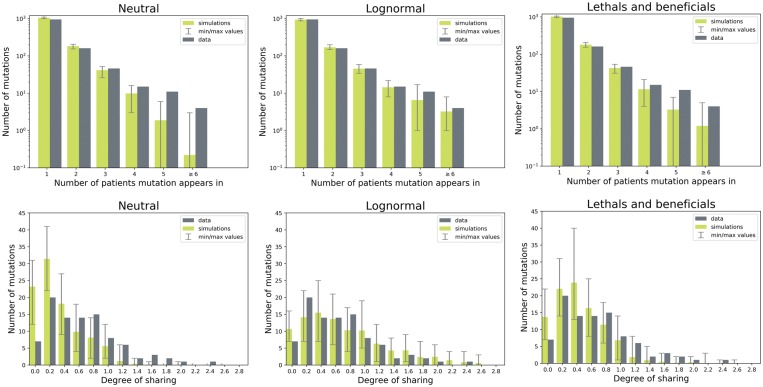
Measures of sharing in the data compared with the mean of 100 simulations: the number of individuals each mutation appears in (top), and the degree of sharing (the average number of individuals the mutations in one subject are shared with, bottom). Left: simulations where mutational effects are neutral; center: simulations where mutational effects are distributed according to the log-normal model; right: simulations where mutations are distributed according to the ‘lethals and beneficials’ model.

The levels of sharing in these two fitted models compared with the data are shown in Fig. 3. Two measures of sharing are shown: the number of individuals each mutation appears in, and the degree of sharing; the average number of individuals the mutations in one individual are shared with. These are the same measures that are used to calculate a subset of the summary statistics that are needed in the ABC-SMC procedure (see Section 4). The simulations from the best-fit models resemble the data much better than the neutral simulations, although the distributions are not perfectly recovered. Interestingly, the log-normal model—which has more deleterious and less beneficial effects produces more convergent evolution than the ‘lethals and beneficials’ model. In the former, the distributions for both measures are shifted to the right compared with the data, indicating higher amounts of sharing, while in the latter, the distribution is slightly shifted to the left compared with the data.

### 2.4 Fitness of shared mutations

**Figure 4.**
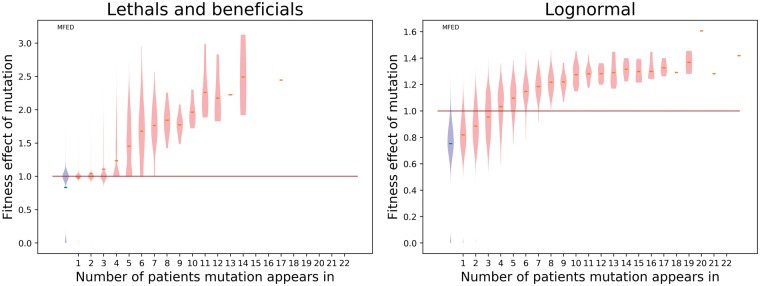
Violin plot representing the distribution of fitness effects per frequency class, derived from hundred simulations using best-fit parameters for both models. The blue violin on the left represents the original MFED, the red lines indicate the median fitness effect in the respective frequency class.

Having an estimate of the MFED and being able to reproduce the datasets with this MFED allows us to estimate the fitness effect of the shared mutations. This can be achieved by finding the average fitness effect of a mutation in a certain frequency class (see Fig. 4). For both models, mutations found in more than four individuals have an MFED with a median higher than one, indicating that this mutation is likely beneficial. The average effect of these mutations differs between the models. If the MFED is distributed according to the ‘lethals and beneficials’ model, the fitness effect of a mutation occurring in fourteen individuals has a fitness effect between 2 and 3 (indicating this mutation will produce two to three times as many offspring per generation). The effect of such a mutation is much lower when the MFED is distributed according to the log-normal model: the effects are then between 1.2 and 1.4.

### 2.5 Time since infection

The TSI of an individual is typically not exactly known, but can be estimated. In the Keele and Li dataset, the TSI has been estimated using both Fiebig stages (Fiebig et al. 2003) and the Poisson estimate by Keele et al. (2008). Fiebig stages are based on clinical data and have a large uncertainty. The Poisson estimate is based on the distribution of inter-sequence hamming distances and a neutral model of evolution.

**Figure 5.**
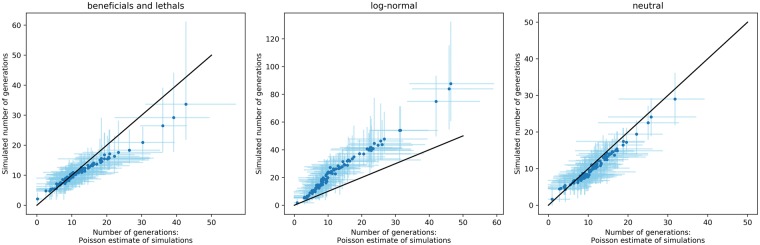
Estimations of TSI for twenty-five simulations of sequence diversification using the best-fit parameters for each model (‘beneficials and lethals’, log-normal and neutral). For each individual in a simulation, the Poisson estimate for TSI was calculated on the final sample, and compared with the true number of generation the simulation ran for. The black line indicates the diagonal, where the real number of generations and the Poisson estimates are the same. The blue dots indicate the mean number of generations for each individual, the light blue lines the minimal and maximal values across all simulations.

The simulations presented here can also be used to directly estimate the TSI, since the simulations run until the number of unique mutations matches the data, rather than fixing the number of generations a simulation should run for. In a neutral model, this estimate should match the Poisson estimate of TSI presented by Keele et al. (2008), where the distribution of inter-sequence hamming distances is assumed to be Poisson-distributed. Indeed, in neutral simulations (see Fig. 5), we see that the Poisson estimate for the number of generations matches the simulated number of generations. However, the presence of fitness effects changes how fast mutations rise and fix in a population, so that the Poisson estimate for TSI might be biased. In general, the presence of beneficial mutations will speed up the accumulation of mutations compared with neutral evolution, causing the Poisson model to overestimate the TSI. Deleterious mutations have the opposite effect and will lead to an underestimation. How a combination of different fitness effects will impact the Poisson estimate depends on the exact number of beneficial and deleterious mutations and is not straightforward to predict. We estimated the TSI using the Poisson method for 25 simulations using the best-fit parameters of the ‘beneficials and lethals’ model and the log-normal model, and compared these estimates with the number of generations that were actually simulated (see Fig. 5). While the two models for the MFED are very similar, the estimates for TSI are affected in different ways. In the ‘beneficials and lethals’ model, the Poisson estimate is a good estimate of the true TSI infection happened recently, but an overestimation if infection occurred more than approximately 10–15 generations (or 20–30 days) before sampling. In the log-normal model, the Poisson estimate is consistently underestimating the real TSI by a factor 2. This is likely due to the many deleterious mutations that take longer to accumulate than neutral mutations.

### 2.6 The role of APOBEC

The mutagenic enzyme APOBEC left a detectable imprint in the data by Keele et al. and Li et al. While APOBEC activity is typically lethal, in some cases its activity is reduced to sub-lethal hypermutation. By studying the sequence context of G-to-A mutations, mutations that were likely due to APOBEC activity can be identified. Of all shared mutations in this dataset, we calculated that 39% are likely APOBEC mediated, while they only make up 12% of all occurring mutations. Nineteen percent of individuals carry APOBEC-mediated mutations. In these individuals, 45% of G-to-A mutations are APOBEC mediated. The distribution of the degree of sharing per subject for the APOBEC individuals is significantly higher from the individuals without any APOBEC mutations (*t*-test, *P*-value 4.74 × 10^–4^, see Supplementary Fig. S1a). However, when correcting for how often G-to-A mutations appear in the dataset (see Supplementary Fig. S1b), they are not more likely to be shared than other mutations, indicating that the higher prevalence of G-to-A mutations due to APOBEC is causing the higher level of sharing, and not the targeting of highly specific motives by APOBEC.

We included the potentially increased mutation rate due to APOBEC into our simulation model because it affects the patterns of sharing. In particular, we assumed that in all individuals that carried APOBEC-mediated mutations the G-to-A mutation rate was increased. We captured this effect in a parameter $\mu_{GA_{APOBEC}}$. This parameter quantifies the increase in the mutation rate in individuals who harbour viruses that are only partially able to counter APOBEC by their vif gene. This parameter should not be confused with the hypermutation rate that APOBEC induces in vif-deleted HIV strains *in vitro* (Kim et al. 2014). Since our parameter $\mu_{GA_{APOBEC}}$ is different from the typical measures of the effect of APOBEC, we cannot directly use estimates from the literature. We therefore estimated the factor by which the G-to-A mutation rate was increased due to non-lethal APOBEC activity ($\mu_{GA_{APOBEC}}/\mu_{GA}$) along with the MFED.

Initially, these estimates were all on the lower end of the prior distribution, which ranged from 1 to 160, while the 95% HPD of the parameter estimate across models ranged from 1 to 34. To make sure, the G-to-A mutation rate increase is significantly different from one, we re-estimated the parameters of the log-normal model with a logarithmic prior distribution for $\mu_{GA_{APOBEC}}/\mu_{GA}$ from $e^{-10}$ to *e*^5^. The estimates remained the same, but the 95% HPD intervals no longer included one, indicating a significant increase in the G-to-A mutation rate due to APOBEC.

## 3. Discussion

The combined datasets of Keele et al. (2008) and Li et al. (2010), containing sequencing data of the *env* gene from over a hundred individuals in early stages of HIV infection, provide many insights into the dynamics of HIV evolution immediately after infection.

Keele et al. (2008), Li et al. (2010) and Lee et al. (2009) found that the pattern of viral diversification in each individual was consistent with a neutral model of viral evolution (the ‘Poisson model’), which they used to estimate the time of infection. However, by considering mutations that are shared across individuals in these datasets, Wood et al. (2009) and Bertels, Metzner, and Regoes (2018) concluded that evolution is not neutral.

In this study, we went one step further than providing evidence that viral evolution is not neutral: we aimed to quantify the fitness effects associated with mutations. In particular, we fitted a global MFED to the sequencing data from Keele et al. (2008) and Li et al. (2010). This approach assumes that the effects of mutations are identical in all individuals, independent of time, environment or sequence context. Our model-fit assigned a high probability to the ‘lethals and beneficials’ and the log-normal model, a low probability to the ‘beneficials only’ and ‘5 spikes’ model, and an almost zero probability to the neutral, ‘lethals only’ and the truncated log-normal model. Due to its high flexibility, we expected the ‘5 spikes’ model to reach higher model probabilities. The low performance is likely due to the high dimensionality of this model (eight parameters vs one to three parameters for the other models), which is inherently punished by the ABC-SMC method (Toni et al. 2009). Although we cannot distinguish between the top two models (‘lethals and beneficials’ and log-normal), their shared characteristics allow us to make several conclusions about the MFED.

Both models contain deleterious and beneficial mutations, indicating that the patterns of sharing cannot only be due to the presence of beneficial mutations. Additionally, the presence of more beneficial mutations does not necessarily mean more sharing, as becomes apparent by comparing the best fits of the log-normal and ‘beneficials only’ models. In the log-normal model, the high amount of deleterious mutations causes higher levels of sharing than the ‘beneficials only’ model, even though the log-normal model contains less beneficial mutations. The presence of deleterious mutations reduces the number of beneficial mutations needed to reach the same level of shared mutations between individuals. Intuitively, this effect is due to an effective shortening of the genome by lethal and deleterious mutations, making sharing by chance more likely. This observation, together with the poor performance of models which do not include deleterious mutations highlights the importance of deleterious mutations when studying convergent evolution on the sequence level.

Among the various types of MFEDs we fitted, the log-normal distribution is closest to MFEDs of other viruses derived from *invitro* studies (Sanjuán, Moya, and Elena 2004; Carrasco, de la Iglesia, and Elena 2007; Domingo-Calap, Cuevas, and Sanjuán 2009; Acevedo, Brodsky, and Andino 2014). According to these studies, 20%–40% of mutations were lethal and 30%–50% deleterious. Beneficial mutations ranged from 0% to 4% of occurring mutations, but small beneficial effects are difficult to detect in competition assays (Eyre-Walker and Keightley 2007). By counting the number of codons that are one mutation away from a stop codon in the consensus *env* gene, we can calculate that 4% of mutations will introduce a premature stop codon, which is typically lethal. This sets a lower bound on the number of lethal mutations, which is just met by the best fit of the log-normal model, for which we found a fraction of lethal mutations of 4.5%. If this model is correct, all lethal mutations in the *env* gene would be due to premature stop codons. It is also important to note that the MFEDs of these other viruses (Sanjuán, Moya, and Elena 2004; Carrasco, de la Iglesia, and Elena 2007; Domingo-Calap, Cuevas, and Sanjuán 2009; Acevedo, Brodsky, and Andino 2014) apply to the entire genome, including non-coding regions. Our estimates of the MFED, in contrast, are based only on the *env* gene, which is under stronger evolutionary pressure than the rest of the genome (Zanini et al. 2015). We therefore expect that the MFED of this gene contains greater deleterious and beneficial effects than the MFED of the whole genome.

The patterns of mutations in individuals have been used to estimate when they became infected by fitting a neutral model of evolution (the ‘Poisson’ model). The existence of beneficial and deleterious mutations that we infer, however, changes the patterns of accumulation of mutations, and hence the estimate of the time of infection. We find that the Poisson model estimates are generally biased when fitness effects are ignored. Interestingly, the direction of the bias depends on the type of the true MFED. If the true MFED is the log-normal model, then the Poisson model consistently underestimates the TSI by a factor of 2. If the ‘beneficials and lethals’ model is correct, then the TSI is overestimated, but only when the TSI is larger than 30 days. While the sequence data do not allow us to differentiate between log-normal and ‘beneficials and lethals’ model, an independent estimate for TSI could help to pin down the type of the true MFED.

The presence of approximately 5% beneficial mutations indicates that a founder virus has opportunities to increase its fitness, and that the respective mutations have not yet fixed globally. Such a situation can arise if there is a trade-off between transmission and within-host replication, and the transmitted founder virus strains will need to ‘re-adapt’ to the within-host environment after transmission. It has been hypothesized for a long time that HIV evolution within the host is ‘short-sighted’ (Levin and Bull 1994; Lythgoe and Fraser 2012; Alizon and Fraser 2013; Fraser et al. 2014; Lythgoe et al. 2017), meaning that adaptation of the virus to the host does not necessarily advance transmission, and might even reduce it. There is empirical evidence that strains from early infection are favored during transmission (Derdeyn et al. 2004; Herbeck et al. 2006). Within-host adaptations that impair transmission include immune escape and reversion, or increased replicative capacity in the secondary lymphatic tissue that may trade-off with replication in anatomical sites important during transmission, such as the lamina propria. There might also be a difference in the fitness effects between chronic and acute infection, since certain sequence motifs are associated with either early or chronic infection (Gnanakaran et al. 2011). This is difficult to differentiate from the differences between transmission and infection; however, as these observed differences might also be caused by early infection still having the motifs of transmission.

The exact sites under strong selection and the selection pressures underlying the MFED cannot be pinpointed with our method. Our inference is based on the fact that there is more sharing of mutations between individuals than is expected by chance. While shared mutations are likely to be more beneficial, our method does not allow the separation of mutations that are shared by chance from those that are shared because they are beneficial. However, other approaches have been able to determine mutations that are likely to be under positive selection (Wood et al. 2009; Bertels, Metzner, and Regoes 2018).

This study also highlights the importance of APOBEC-mediated hypermutation in HIV evolution. The protein is part of the hosts’ innate immune response against viruses that mutagenizes single-stranded DNA (Goila-Gaur and Strebel 2008). Considering that, according to the MFEDs estimated here, 20%–95% of mutations will be deleterious or lethal, an increased number of mutations will likely reduce fitness of the virus, thereby limiting viral spread. However, there is also a small chance of introducing beneficial mutations, which via selection will rapidly fix in the population. Selection of APOBEC-mediated mutations has been observed in HIV before (Wood et al. 2009; Kim et al. 2014) and our study suggests the same. Virus samples carrying APOBEC-mediated mutations show higher levels of sharing than those without APOBEC signature. This higher level of sharing could arise because APOBEC-mediated mutations occur at a limited fixed set of sites, or because APOBEC increases the mutation rate and thus speeds up evolution and fixation of beneficial mutations. The collective evidence in our study suggests that APOBEC increases sharing by increasing the mutation rate (see Supplementary Fig. S1).

The concept of a global MFED has limitations. The first limitation is that differences between hosts cannot be captured by a global MFED. Genetic differences between hosts may lead to only subtle deviations from the assumption of universal fitness effects, while strain-specific immune responses result in large changes in fitness effects that are tied to a specific host at a specific time. In general, immune pressure, for example from cytotoxic T-lymphocytes (CTL) is considered the most important source of selection in HIV infection. Therefore, we relied on data sampled in early infection, when immune responses have not yet been fully mounted and when the assumption of fitness effects that apply in every environment and are constant is still plausible. Although CTL escape can happen very early in HIV infection, there is still a period of time where CTL escapes are rarely observed. Goonetilleke et al. (2009), for example, monitored three HIV positive individuals from the moment of diagnosis, in all cases in Fiebig stage II. No CTL escapes were found at the moment of diagnosis, the first escapes arose within 10–20 days after diagnosis. The majority of individuals in the dataset, we used were also sampled in Fiebig stage II. This means that the majority of the individuals used in this study were sampled at a time early enough for immune escape to be unlikely. Approximately 30 individuals were sampled at later Fiebig stages, at which time immune escape is more common. It is likely that some of the mutations in these individuals are selected by an immune response. However, in order to have an impact on our analysis, these mutations must contribute significantly to sharing. In order for an escape mutation to be shared, it is not enough that the hosts share HLA alleles, but the virus needs to pick up the exact same escape mutation. If we look at the escape mutations identified by Wood et al. (2009) in the Keele & Li dataset, the majority of escape mutations (a total of twenty-three escape mutations were identified at six epitopes) are present in only one or two individuals, with only a single escape mutation present in more than two individuals. Reversion of CTL escape variants in the previous host could also confound our analysis. However, reversion is even less likely than escape (Fryer et al. 2010), and Wood et al. only found two sites that were identified as reversion mutations.

The concept of an MFED also fails to account for a potentially changing fitness landscape due to changes in the anatomical environment. In fact, HIV is not replicating in a constant environment from the moment of infection. After transmission, HIV migrates via different tissues before establishing a reservoir in the secondary lymphoid organs (Haase 2005). Each tissue might affect the fitness of the virus differently. Since our dataset contains samples from one to several weeks after infection, and establishment of the reservoir typically happens after the first week of infection (Haase 2005), the MFED we estimated might represent an average of the MFED in the different tissues. Also, frequency-dependent selection cannot be captured by an MFED. In HIV infection, frequency-dependent selection is exerted by strain-specific adaptive immune responses that target the most common strains. This type of frequency-dependent selection will be more prominent in later stages in infection when adaptive immune responses are fully mounted. However, we cannot exclude that there are other, unknown, sources of frequency-dependent selection present in early infection.

Additionally, our approach to estimate the MFED neglects epistasis because we assume that the effects of mutations simply multiply. But again, we can mitigate the severity of this assumption by using data from early infection, when the number of mutations per sequence is expected to be low. Interactions between mutations are therefore unlikely.

Despite all these caveats, the MFED is a useful concept worth of study (Eyre-Walker and Keightley 2007; Sanjuán 2010). Because the effect of a mutation is sensitive to the environment, compiling a comprehensive table of the fitness effects of all mutations (a ‘fitness landscape’) in every relevant environment is out of reach for most systems. While the global MFED does not pinpoint the exact locations of mutations with advantageous or disadvantageous effects, it provides a way to average over the variation in mutational effects and thus provides insights into the adaptive potential of an organism. By estimating the MFED from clinical data, our study captures the adaptive potential of HIV-1 relevant in its epidemiological setting.

## 4. Methods

### 4.1 Data and identification of shared mutations

For all of the individuals from Keele et al. (2008) and Li et al. (2010), a sequence sample and a consensus sequence are available. Only the sequences from the individuals in which the infection was the result of a single founder virus were used. These were all aligned to a reference sequence HIV-1 NL-43 (Giallonardo et al. 2014). Mutations in two individuals are considered shared if they map to the same position in the reference genome and have the same base in the consensus sequence that changed into the same mutated base. Deletions and insertions were not considered. An overview of all used individuals, including GENBANK accession numbers can be found in Supplementary Table S2.

Sequences from this dataset where mutations carrying an APOBEC signature are removed using hypermut 2.0 (Rose and Korber 2000) were acquired from Elena Giorgi. These are the sequences also used in Wood et al. (2009). Any mutations that do not occur in these sequences, but are present in the original sequences, were identified by Hypermut 2.0 and are thus considered APOBEC-mediated mutations.

### 4.2 Simulations of sequence evolution

Simulations of sequence evolution were adapted from Lee et al. (2009). The first step of the sequence evolution simulation is the initialization, which consists of ancestor sequence generation and the initialization of the fitness table. The sequence generation is the same as in Lee et al. and results in a random sequence, *s*, with the same length *N* and base distribution as the *env* gene. For this sequence, a fitness table can then be created. This is a table of size *N* by 4, where each entry is the fitness effect of each possible base at every position. The entries for the ancestor sequence are set to 1, the rest is filled up with random draws from the MFED.

During the simulation, each sequence in every generation generates offspring according to Poisson $\left(R_{0}*f\right)$, where *f* is the total fitness of the sequence, as

$$f=\prod_{i}^{N}F[s[i],i]$$

where *F* is the fitness table, *s* is the sequence, and *i* is the position of the sequence. F[s[i],i] is the fitness value of the base at position *i* in sequence *s*. This is the product of the entries in the fitness table for all positions in the sequence. Every new offspring sequence acquires new mutations according to mutation rate *μ* and substitution matrix *M*.

In the simulations, new mutations are selected as follows. First, the number of mutations that will happen on the sequence is decided by drawing from a Poisson distribution with mean *μ*. Then, a position is randomly chosen to mutate into another random base. This mutation is then accepted or rejected based on the transition matrix M, making some mutations more likely than others. New mutations are proposed until the desired number of mutations have been accepted. The transition matrix we use was calculated by Lee et al. (2009) from a maximum likelihood general time reversible (GTR) model of substitutions that occur in the full length HIV-1 envelope gene. However, we have found that using different definitions of the mutation matrix does not affect the parameter estimates. Optionally, the G-to-A mutation rate can be increased *x*-fold to simulate APOBEC-mediated G-to-A hypermutation.

The population is capped to 10,000 sequences. Once the number of sequences exceeds this cap, 10,000 sequences are randomly sampled from the population and used as the next generation. The population cap imposed here is in line with some estimated of the effective population size of HIV (Kouyos, Althaus, and Bonhoeffer 2006; Rouzine and Weinberger 2013). To make sure our maximum number of sequences is sufficient, we have tested a subset of the fits with a higher maximum capacity of 10^5^. Parameter estimates remained the same, indicating that a maximum capacity of 10^4^ is sufficient for this application.

In order to recreate the dataset, a separate simulation is performed for each individual with the same initial sequence and fitness table. After each generation, a sample is taken from the simulation with the same size as the number of sequences available for the individual. The number of unique mutations in this sample is then counted. If this matches the data, the simulations are stopped and this sample is used for analysis. If this number exceeds the number of mutations present in the data, the previous sample is also compared and the sample with the closest number of mutations is used.

The simulations of sequence evolution were implemented in python. The code is available at https://gitlab.ethz.ch/bonse/MFED/.

### 4.3 Approximate Bayesian computation using sequential Monte Carlo

We defined several different models for the parametrization of the MFED (see Section 2). The ABC-SMC framework (standing for approximate Bayesian computation using sequential Markov chains Toni et al. (2009)) was then used for simultaneous model selection and parameter estimation of the MFED. For this, we implemented an SMC procedure in python. The code is available at https://gitlab.ethz.ch/bonse/MFED/.

The fitting procedure starts with equal probabilities for all models. Simulations are then performed for all models with random parameters according to their prior distribution. The priors were uniform distributions from 0 to 1 for all parameters except λ (0,2) in the exponential beneficials models, μ (−1,1) in the log-normal models and $b_{b}\;(1,2)$ in the 5 spikes model. In the first iteration, a set of parameters (a ‘particle’) is sampled and a simulation is run with these parameters. If the distance between the summary statistics of this simulation and the data is smaller than *ϵ*_1_, the particle is retained. Once 1,000 particles have been accepted, a weight is assigned to all accepted particles (Toni et al. 2009).

In the next generations, a particle is sampled from the previous iteration using the assigned weights. The particles are then perturbed according to Gaussian kernel and a simulation is run with these parameters. Then, a set of summary statistics (see later) is calculated. If the distance between these summary statistics and the data is smaller than *ϵ_i_* (with *i* the iteration), the particle is retained and once 1,000 particles have been accepted, the weights are calculated.

We used tolerances $\epsilon_{i}=[2.2,1.3,0.8,0.7,0.6,0.5,0.4]$, and a Gaussian kernel with *σ* equal to the average distance between accepted particles in the model divided by 2. We used a normalized Euclidean distance to calculate the distance between simulations and the data, where the summary statistics are divided by the corresponding summary statistics of the data, after which the distance to a vector of ones is calculated.

The model probabilities are directly calculated from the set of accepted parameters (the number of accepted particles for model *x*/1,000). It is possible for a model to ‘die out’ during the SMC if none of the particles for this model are accepted. This indicates an exceedingly low model probability.

For the parameter estimation, we did a Gaussian kernel density estimation for the accepted parameters in the SMC in the last iteration for each remaining model. From this, we determined the highest density point—which was used as the parameter estimate—and the 95% highest density intervals.

### 4.4 Summary statistics

In total, fourteen summary statistics were defined to calculate the distance between simulations in data. The majority of them are based on shared mutations, which are defined as a mutation that occurs in the same position with the same from- and to-base in two independent individuals.

Five of the summary statistics are based on the average degree of sharing (*s_x_*) of the mutations in an individual *x*. This is an individual-centered metric calculating the average number of other the mutations in *x* appear in, and is defined as:

$$s_{x}=\frac{\sum_{i=0}^{m}c_{ix}}{m}$$

where *m* is the number of mutations in individual *x* and $c_{i,x}$ is the number of individuals other than *x* that carry mutation *i*.

This number can be calculated for all ninety-eight individuals in the dataset. The resulting distribution is used to calculate several summary statistics: the mean and the median of the distribution, the difference between mean and median, the variance of the distribution and the area under the cumulative curve of the sorted degrees of sharing per individual.

Where the degree of sharing characterizes sharing for each individual, the occurrence of mutations is a similar measure, this time calculated per mutation. It measures in how many individuals each occurring mutation is present. These numbers are then sorted and categorized. For the summary statistics, the following five categories are used: the number of mutations occurring only once (singletons), the amount occurring in exactly two individuals, those occurring three to five times, five to ten times and finally the number of mutations occurring in more than ten individuals.

The remaining four summary statistics are individual based, and three serve as a proxy to match the number of generations to the data. They are calculated for each individual separately, and averaged for the summary statistic. A first measure is the fraction unmutated sequences, the percentage of sequences in the sample that is identical to the founder. For the simulations the founder is known, for the data the consensus sequence is assumed to be the same as the founder sequence. The fraction unmutated is then used to also calculate a selection index, the percentage of sites in the sample that are mutated in at least one sequence, divided by the percentage of mutated sequences in the sample (which is 1 – %unmutated). Lastly, the average inter-sequence hamming distance is calculated per viral population, which, according to Lee et al. (2009), is directly related to the TSI in a neutral model.

In order to match the G-to-A mutation rate increase as well, the fraction of G-to-A mutations in the sample was calculated per individual. The mean of this distribution was then used as the last summary statistic.

## Funding

We gratefully acknowledge the financial support from the Swiss National Science Foundation (grant number 31003A_149769 to RRR).

**Conflict of interest**: None declared.

## Supplementary Material

Supplementary Data S1

Supplementary Data S2

Supplementary Data S3
