# Supplementary material for: Estimating the mutational fitness effects distribution during early HIV infection
Source: Virus Evol. 2018 Oct 4;4(2):vey029. doi: 10.1093/ve/vey029 (PMC6172364; doi:10.1093/ve/vey029)
Supplement: Supplementary Data S1 [file vey029_supp_s1.pdf]

(a)

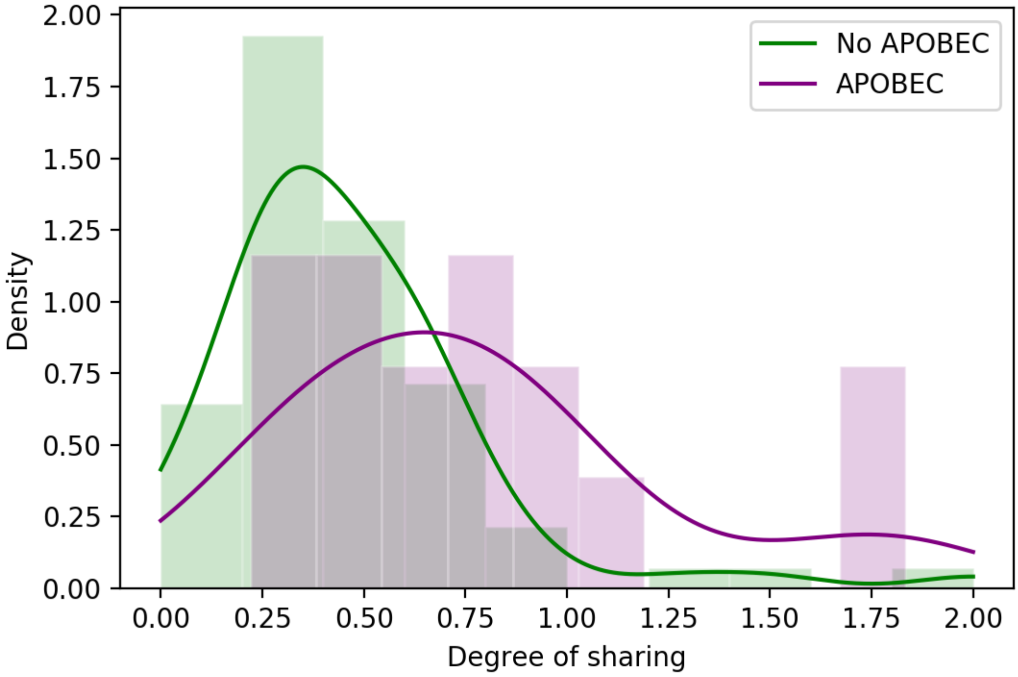

(b)

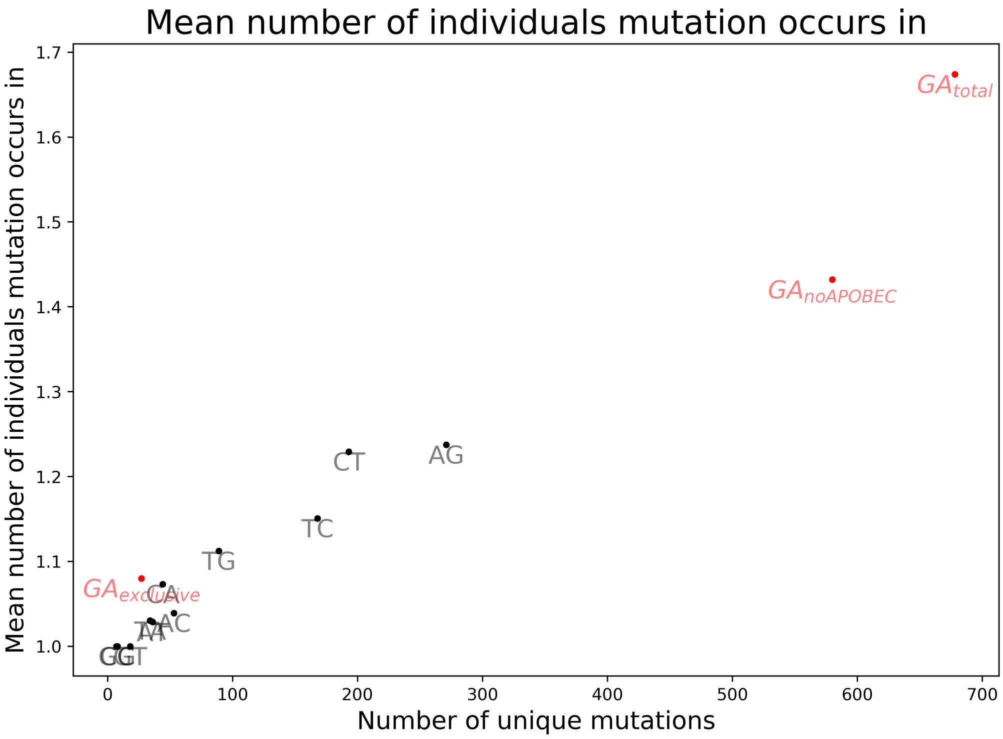

Figure S1: (a) distribution of the shared index for individuals with (purple) and without (green) APOBEC-mediated mutations. The individuals carrying APOBEC-mediated mutations have a significantly higher degree of sharing than those not having any mutations with APOBEC signature. (b) The mean number of individuals a mutation occurs in vs. the number of occurrences for each 'type' of mutation. The gray points represent all mutations that were not APOBEC-mediated. The red points indiate G-to-A mutations. $Ga_{noAPOBEC}$ excludes all apobec-mediated mutations, $GA_{exclusive}$ are G-to-A mutations that are exclusively APOBEC-mediated, GA_{total} combines all G-to-A mutations. APOBEC introduces many mutations, but these mutations are not more shared than other mutations when correcting for their prevalence.
