# Supplementary material for: Estimating the mutational fitness effects distribution during early HIV infection
Source: Virus Evol. 2018 Oct 4;4(2):vey029. doi: 10.1093/ve/vey029 (PMC6172364; doi:10.1093/ve/vey029)
Supplement: Supplementary Data S2 [file vey029_supp_s2.pdf]

ID	GENBANK accession	APOBEC	source	number of sequences	number of unique mutations
63358	EU575707-EU575733	yes	Keele et al.	27	24
1001	EU574937-EU574998	yes	Keele et al.	62	44
4013440	GU330809-GU330838	no	Li et al.	30	14
1006	EU574999-EU575040	no	Keele et al.	42	12
4013446	GU330839-GU330861	no	Keele et al.	23	15
PRB931	EU576568-EU576586	no	Keele et al.	19	20
SC51	EU577006-EU577038	no	Keele et al.	32	13
TRJO4551	EU577101-EU577118	no	Keele et al.	16	12
MEMI4948	EU576522-EU576553	no	Keele et al.	32	44
701010108	GU331183-GU331217	no	Keele et al.	35	30
700010077	GU330916-GU331038	no	Keele et al.	53	31
Z05	EU577447-EU577461	no	Keele et al.	15	10
Z36	EU577762-EU577778	no	Keele et al.	17	17
Z02	EU577404-EU577424	no	Keele et al.	21	13
Z34	EU577723-EU577740	no	Keele et al.	18	10
TT34P	EU577272-EU577300	no	Keele et al.	29	13
Z33	EU577702-EU577722	no	Keele et al.	21	16
Z32	EU577692-EU577701	no	Keele et al.	10	5
Z31	EU577675-EU577691	no	Keele et al.	17	31
4013242	GU330462-GU330498	no	Keele et al.	37	29
63215	EU575688-EU575706	yes	Keele et al.	18	8
62357	EU575499-EU575512	no	Keele et al.	14	7
WEAU0575	EU577344-EU579293	no	Keele et al.	43	33
SC45	EU576977-EU577005	no	Keele et al.	29	13
Z13	EU577479-EU577509	no	Keele et al.	30	40
REJO4541	EU576706-EU576726	no	Keele et al.	21	13
Z27	EU577604-EU577628	no	Keele et al.	25	22
Z20	EU577563-EU577588	no	Keele et al.	26	14
Z23	EU577589-EU577603	no	Keele et al.	15	6
TT35P	EU577301-EU577343	yes	Keele et al.	42	21
62995	EU575621-EU575647	no	Keele et al.	26	10
9028	EU576027-EU576049	no	Keele et al.	23	5
9029	EU576050-EU576071	yes	Keele et al.	21	10
9023	EU575950-EU575967	no	Keele et al.	18	12
9024	EU575968-EU575992	no	Keele et al.	23	14
9025	EU575993-EU576011	no	Keele et al.	19	9
SC31	EU576889-EU576924	no	Keele et al.	36	29
9020	EU575868-EU575892	no	Keele et al.	25	14
9021	EU575893-EU575926	no	Keele et al.	34	7
9022	EU575927-EU575949	yes	Keele et al.	22	8
PRB926	EU576554-EU576567	no	Keele et al.	14	10
62130	EU575488-EU575498	no	Keele et al.	11	6
SC22	EU576851-EU576888	no	Keele et al.	38	23
THRO4156	EU577074-EU577100	no	Keele et al.	27	13
1058	EU575329-EU578828	yes	Keele et al.	45	35
SC20	EU576808-EU576850	no	Keele et al.	40	14
1059	EU575374-EU578926	yes	Keele et al.	39	38
1056	EU575283-EU578774	no	Keele et al.	46	15
9032	EU576111-EU576149	no	Keele et al.	39	11
9033	EU576150-EU576169	no	Keele et al.	20	12
9030	EU576072-EU576090	no	Keele et al.	18	11
9031	EU576091-EU576110	yes	Keele et al.	20	15
701010055	GU331066-GU331093	no	Keele et al.	28	11
9079	EU576248-EU576273	no	Keele et al.	26	11

INME0632	GU331676-GU331721	no	Keele et al.	46	23
RHPA4259	EU576727-EU576757	yes	Keele et al.	31	25
63396	EU575734-EU575754	no	Keele et al.	21	4
SC11	EU576788-EU576807	no	Keele et al.	19	1
9075	EU576170-EU576191	no	Keele et al.	22	5
9077	EU576224-EU576247	no	Keele et al.	23	9
1054	EU575244-EU575282	no	Keele et al.	36	14
1053	EU575184-EU575243	yes	Keele et al.	57	20
61792	EU575469-EU575487	no	Keele et al.	18	7
700010246	GU330994-GU331038	yes	Keele et al.	45	28
SC05	EU576758-EU576787	no	Keele et al.	29	13
700010106	GU330916-GU330955	yes	Keele et al.	40	28
TT31P	EU577205-EU579134	no	Keele et al.	67	31
AD75	GU331496-GU331549	yes	Keele et al.	54	27
9010	EU575755-EU575773	no	Keele et al.	19	10
6248	EU575573-EU575592	yes	Keele et al.	20	24
9015	EU575788-EU575823	no	Keele et al.	36	11
9014	EU575774-EU575787	no	Keele et al.	13	4
9019	EU575850-EU575867	no	Keele et al.	17	9
9017	EU575824-EU575849	no	Keele et al.	26	6
700010040	GU330916-GU331038	no	Keele et al.	30	15
4013291	GU330499-GU330523	no	Keele et al.	25	19
HOBR0961	GU331634-GU331675	no	Keele et al.	42	15
TT29P	EU577185-EU577204	no	Keele et al.	20	5
4013296	GU330524-GU330548	no	Keele et al.	25	20
6240	EU575513-EU575529	no	Keele et al.	17	8
WITO4160	EU577388-EU577403	no	Keele et al.	16	11
4013396	GU330692-GU330730	no	Keele et al.	39	17
6244	EU575530-EU575540	no	Keele et al.	11	4
6247	EU575541-EU578951	no	Keele et al.	31	7
701010027	GU331039-GU331065	no	Keele et al.	27	18
700010058	GU330916-GU331038	no	Keele et al.	47	38
4013327	GU330598-GU330621	no	Keele et al.	24	10
4013321	GU330549-GU330597	no	Keele et al.	49	36
4013226	GU330363-GU330395	no	Keele et al.	33	17
TT28P	EU577157-EU577184	no	Keele et al.	28	9
1018	EU575084-EU575133	yes	Keele et al.	50	40
1012	EU575041-EU575083	no	Keele et al.	42	23
63054	EU575648-EU575667	no	Keele et al.	20	9
SUMA0874	EU577039-EU579128	yes	Keele et al.	35	11
12007	EU575413-EU575437	yes	Keele et al.	25	17
PRB956	EU576587-EU576613	no	Keele et al.	27	12
PRB958	EU576650-EU576673	no	Keele et al.	24	11
PRB959	EU576674-EU576705	no	Keele et al.	32	12

Table S2: Accession numbers, extracted statistics and original source of the sequences used to estimate the MFED.
