# Supplementary material for: Estimating the mutational fitness effects distribution during early HIV infection
Source: Virus Evol. 2018 Oct 4;4(2):vey029. doi: 10.1093/ve/vey029 (PMC6172364; doi:10.1093/ve/vey029)
Supplement: Supplementary Data S3 [file vey029_supp_s3.pdf]

a.

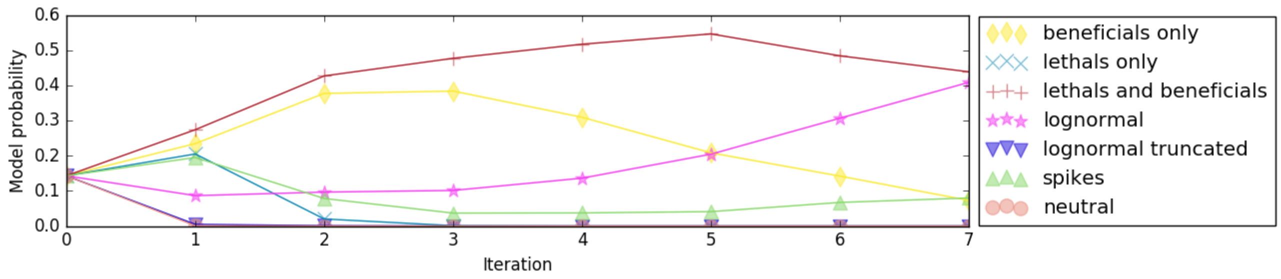

b.

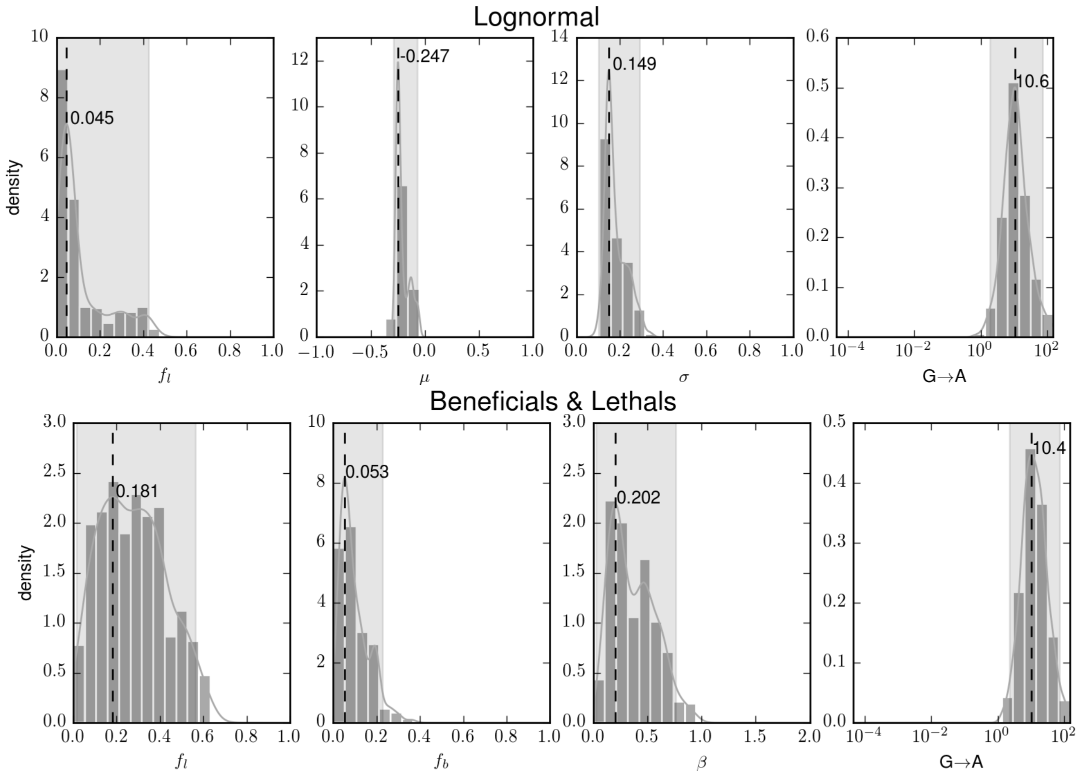

Figure S3: Results of the ABC-SMC fitting procudure. (a) probabilities of the different models S3 across iterations. (b) posterior distribution of the parameters for the lognormal model (top) and ‘beneficials and lethals’ model (bottom). Grey shaded areas indicate the 95% HPD intervals, the black dotted line the highest density point (which is used as the best estimate for the parameters)
